# Postoperative changes of the microbiome: are surgical complications related to the gut flora? A systematic review

**DOI:** 10.1186/s12893-017-0325-8

**Published:** 2017-12-04

**Authors:** Ann-Kathrin Lederer, Przemyslaw Pisarski, Lampros Kousoulas, Stefan Fichtner-Feigl, Carolin Hess, Roman Huber

**Affiliations:** 1 Center for Complementary Medicine, Department of Environmental Health Sciences and Hospital Infection Control, Medical Center – University of Freiburg, Faculty of Medicine, University of Freiburg, Breisacher Straße 115b, 79106 Freiburg im Breisgau, Germany; 2 Department for General and Visceral Surgery, Medical Center – University of Freiburg, Faculty of Medicine, University of Freiburg, Freiburg, Germany; 3 Department for Thoracic Surgery, Medical Center – University of Freiburg, Faculty of Medicine, University of Freiburg, Freiburg, Germany

**Keywords:** Microbiota, Gastrointestinal microbiome, Postoperative complications, Anastomotic leakage, Surgical wound infection

## Abstract

**Background:**

The purpose of this review was to identify the relationship between the gut microbiome and the development of postoperative complications like anastomotic leakage or a wound infection. Recent reviews focusing on underlying molecular biology suggested that postoperative complications might be influenced by the patients’ gut flora. Therefore, a review focusing on the available clinical data is needed.

**Methods:**

In January 2017 a systematic search was carried out in Medline and WebOfScience to identify all clinical studies, which investigated postoperative complications after gastrointestinal surgery in relation to the microbiome of the gut.

**Results:**

Of 337 results 10 studies were included into this analysis after checking for eligibility. In total, the studies comprised 677 patients. All studies reported a postoperative change of the gut flora. In five studies the amount of bacteria decreased to different degrees after surgery, but only one study found a significant reduction. Surgical procedures tended to result in an increase of potentially pathogenic bacteria and a decrease of Lactobacilli and Bifidobacteria. The rate of infectious complications was lower in patients treated with probiotics/symbiotics compared to control groups without a clear relation to the systemic inflammatory response. The treatment with synbiotics/probiotics in addition resulted in faster recovery of bowel movement and a lower rate of postoperative diarrhea and abdominal cramping.

**Conclusions:**

There might be a relationship between the gut flora and the development of postoperative complications. Due to methodological shortcomings of the included studies and uncontrolled bias/confounding factors there remains a high level of uncertainty.

**Electronic supplementary material:**

The online version of this article (10.1186/s12893-017-0325-8) contains supplementary material, which is available to authorized users.

## Background

Every human being lives in a sensitive balance with an amount of different microorganisms [1–4]. The constitutional symbiosis between the host and his microbiome suggests that there is a relationship regarding human health. A variety of diseases like Crohn’s disease and Ulcerative Colitis are nowadays known to be associated with the gut microbiome [5, 6]. The microbiome is influenced by various factors like psychological stress, the circadian rhythm and cultural as well as ethnical factors [2, 7]. Also eating habits affect the microbiome, especially the flora of the gut [8, 9]. The technical progress of the last decades allows rapid and reliable gut microbiome analysis with next generation sequencing [3]. Therefore, it was possible to get new insights into the microbiological spectrum of the gut. The flora of the gut is dominated by strains of Firmicutes and Bacteroidetes [2, 3]. Potentially pathogenic bacteria like Pseudomonas, Enterobacteriacae like Escherichia and Klebsiella, Enterococcus and Staphylococcus also occur in the normal gut flora, but only in small quantities [2, 6]. Recent literature suggests that these bacteria might be involved in development of infectious complications after gastrointestinal surgery [2, 10, 11]. Infectious complications are a leading problem after gastrointestinal surgery and are associated with high mortality and morbidity rates [12, 13]. Despite addressing the established risk factors for gastrointestinal surgical complications by e. g. improved operation techniques, the published rates of anastomotic leakage and wound infections remain a relevant problem and did not change in the past years. It strongly varies (3–30% for anastomotic leakage) between hospitals and patients groups and the cause of this variation is widely unknown [12, 14]. The above-mentioned influence of the gut flora might be more important than previously expected. Recent reviews found a relationship between the development of an anastomotic leakage and the gut flora [1, 15–17]. These reviews however focused on preclinical data and did not comprehensively consider the clinical evidence. Consequently, it is necessary to review the current literature from a clinical perspective. In respect to the advance of science this review summarizes the current literature and intends to highlight the relationship between the gut flora and the development of postoperative complications after surgical procedures on the gastrointestinal tract.

## Methods

### Aim of the review and search strategy

The hypothesis of this systematic review was that the gastrointestinal microbiome of patients, who developed postoperative complications like anastomotic leakage or wound infection, differed from patients with an uncomplicated postoperative course.

The systematic research was performed in January 2017. Last day of search was the 30th of January 2017. We used the data bases of Medline and WebOfScience (see the Additional file 1 for hyperlinks). There was no language restriction. Search terms were selected in English (see the Additional file 1 for the whole search strategy). There were three main terms (“gut microbiome”, “gut microbiota” and “intestinal flora”), which were refined by four additional terms (“wound healing”, “wound infection”, “postoperative complications” and “anastomotic leak”). In addition reference lists of included studies were screened by title and abstract for eligible publications.

### Collection and evaluation of data

Search and screening by abstract and title was realized by AKL and RH in English and German. Polish publications were screened and reviewed by PP, French ones by CH. Full text screening and data extraction was finally performed by AKL and RH manually. Data was collected in a predesigned table. Selected characteristics for extraction were constitution of gut microbiome, method of quantification, duration and kind of applied synbiotics/probiotics, occurrence of infections and nutritional and antibiotic treatment pre- and postoperatively (all parameters are shown in Tables 1, 2 and 3). Risk of bias was evaluated by methodological quality of studies (sample size calculation, definition of primary endpoints, criteria of inclusion and exclusion, completeness of outcome data and additional for RCTs blinding, comparability of groups and treatment with placebo). Searching and screening were performed non-blinded, but in accordance with the Cochrane guidelines.

**Table 1:** Probiotics and synbiotics in the included RCTs

Author	Preparation	Duration of application
Kanazawa et al.	Bifidobacterium breve Lactobacillus casei Galactooligosaccharides	Start: 1 day postoperative Stop: 14 days postoperative
Reddy et al.	Lactobacillus acidophilus Lactobacillus bulgaris Bifidobacterium lactis Streptococcus thermophiles Oligofructose	Start: 1 day preoperative Stop: Postoperative, day not reported
Sugawara et al.	Bifidobacterium breve Lactobacillus casei Galactooligosaccharides	Start: 14 days preoperative Stop: 14 days postoperative
Liu et al.	Lactobacillus plantarum Lactobacillus acidophilus Bifidobacterium longum	Start: 6 days preoperative Stop: 10 days postoperative
Eguchi et al.	Bifidobacterium breve Lactobacillus casei Galactooligosaccharides	Start: 2 days preoperative Stop: 14 days postoperative
Usami et al.	Bifidobacterium breve Lactobacillus casei Galactooligosaccharides	Start: 14 days preoperative Stop: 14 days postoperative
Zhang et al.	Bifidobacterium longum Lactobacillus plantarum Enterococcus faecalis	Start: 5 days preoperative Stop: 2 days preoperative
Okazaki et al.	Bifidobacterium breve Lactobacillus casei Galactooligosaccharides	Start: 7 days preoperative Stop: 10 days postoperative
Tanaka et al.	Bifidobacterium breve Lactobacillus casei Galactooligosaccharides	Start: Preoperative, day not reported Stop: 21 days postoperative
Tanaka et al. (Control group)	Streptococcus faecalis	Start: 1 day postoperative Stop: 21 days postoperative

**Table 2:** Summary of included publications

Author	Kanazawa et al.	Reddy et al.	Sugawara et al.	Liu et al.	Eguchi et al.
Year	2005	2006	2006	2010	2010
Country	Japan	UK	Japan	China	Japan
Study type	RCT	RCT	RCT	RCT	RCT
Treatment	Synbiotics vs. no Treatment	*	**	Probiotic vs. Placebo	Synbiotics vs. no Treatment
Number of patients included	44 (21 + 23)	88 (24 + 22 + 20 + 22)	81 (41 + 40)	100 (50 + 50)	50 (25 + 25)
Area of surgery	Liver	Colon	Liver	Colon	Liver (Transplant)
Method of quantification	Culture	PCR	Culture	PCR	Culture
C-reactive Protein	Recovery faster	No difference	↓	n. s.	n. s.
White blood cell count	Recovery faster	n. s.	↓	n. s.	n. s.
SIRS	n. s.	No difference	n. s.	No difference	n. s.
Organic acids in stool	↑	n. s.	↑	n. s.	n. s.
Stool pH	No difference	n. s.	No difference	n. s.	n. s.
Rate of infections	↓	No difference	↓	↓	↓
Number of pathogenetic bacteria	↓	↓	No difference	↓	No difference
Number of Lactobacilli and Bifidobacteria	↑	n. s.	No difference	↑	n. s.

If not specified elsewhere, the arrows show the postoperative comparison of the synbiotic/probiotic-treated patients compared to control groups

*n. s.* not specified, *RCT* Randomized controlled trial, *SIRS* Systemic inflammatory response syndrome

*Group 1: Mechanical bowel preparation (MBP) only, Group 2: MBP + Neomycin, Group 3: MBP + Neomycin + Synbiotics, Group 4: Synbiotics + Neomycin, no MBP

**Preoperative synbiotic treatment vs. no treatment, both groups were treated with synbiotics postoperatively

**Table 3:** Summary of included publications

Author	Usami et al.	Zhang et al.	Okazaki et al.	Ohigashi et al.	Tanaka et al.
Year	2010	2012	2013	2013	2012
Country	Japan	China	Japan	Japan	Japan
Study type	RCT	RCT	RCT	Cohort	RCT
Treatment	Synbiotics vs. no Treatment	Probiotics vs. Placebo	Synbiotics vs. no Treatment	n. s.	Synbiotics vs. Probiotics
Number of patients included	61 (29 + 32)	60 (30 + 30)	48 (25 + 23)	81	64 (30 + 34)
Area of surgery	Liver	Colon	Pankreas/Bile-System	Colon	Oesophagus
Method of quantification	Culture	Culture	PCR	PCR	PCR
C-reactive Protein	No difference	↓	No difference	n. s.	↓
White blood cell count	↓	n. s.	No difference	n. s.	↓
SIRS	↓	n. s.	↓	n. s.	↓
Organic acids in stool	↓	n. s.	↑	↓ ^a^	↑
Stool pH	↓	n. s.	↓	No difference	↓
Rate of infections	↓	↓	↓	7,4%	↓
Number of pathogenetic bacteria	↑	↓	↓	↑ ^a^	↓
Number of Lactobacilli and Bifidobacteria	↓	↑	↑	↓ ^a^	↑

If not specified elsewhere, the arrows show the postoperative comparison of the synbiotic/probiotic-treated patients compared to control groups

*n. s.* not specified, *RCT* Randomized controlled trial, *SIRS* Systemic inflammatory response syndrome

^a^Compared to preoperative measures

### Predefined in- and exclusion criteria

We decided to focus on the clinical perspective and therefore included studies of all type performed in humans only. Studies with all kind of patients (no age or gender limitation) and all kind of surgical procedures of the gastrointestinal tract were included. The examination of stool was an obligatory inclusion criterion. Stool examination had to be done by culture or sequencing (polymerase chain reaction). Application of different perioperative antibiotics within a trial was a criterion for exclusion. Studies without relation to a surgical procedure, investigating wounds, which were caused by toxic damage or burning, or only focusing on long-term consequences after surgery, were excluded. To meet criteria of inclusion infectious complications were supposed to be recorded for up to 30 days postoperatively or until discharge. These were supposed to be directly related to the performed operation. Unpublished material, meta-analyses and reviews were not considered.

### Definition of complications

The total number of infectious complications resulted from summation of reported incidence of pneumonia, wound infection, intraabdominal abscess, urinary tract infection, anastomotic leakage and septic morbidity. Pneumonia was defined as a combination of characteristic pulmonary infiltrates on chest x-ray accompanied by leukocytosis. Spontaneous or surgically drained purulent discharge of a wound was defined as a wound infection. An intraabdominal fluid collection with requirement of drainage was defined as an intraabdominal abscess. An anastomotic leakage was diagnosed radiologically (leakage of contrast medium) or endoscopically (visible defect of anastomosis). Sepsis and SIRS were defined according to the consensus definition of critical care medicine [18].

## Results

### Descriptive characteristics

**Fig. 1:**
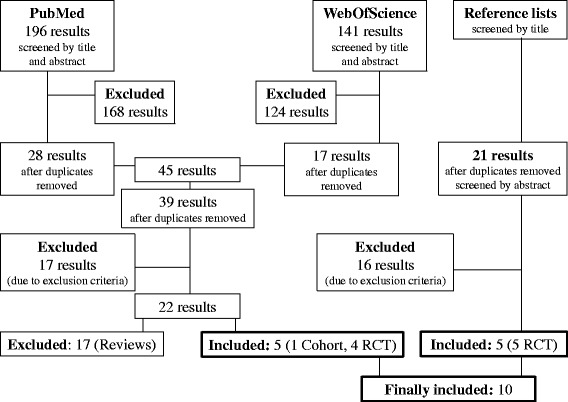
Process of screening and selection

According to the predefined in- and exclusion criteria we identified 196 results with Medline and 141 results with WebOfScience. After screening by title and abstract and removing the duplicates there were 39 remaining results. We did a full text screening and excluded further 17 publications because of missing stool examination, application of different antibiotics perioperatively, no relation to surgery or adressing long-term consequences like pouchitis after restorative proctocolectomy [19–34]. Twenty-two publications remained, but 17 of them were reviews, which were finally excluded. Additional five publications could be added after screening reference lists of included studies [35–39]. The whole process of data base screening and selection is shown in Fig. 1. In total ten publications were included. Results of the included studies are summarized in Tables 2 and 3.

From the ten studies nine were performed in Asia (Japan and China) and one in the United Kingdom. Publications were released between 2006 and 2012. One study is related to a cohort study (*n* = 81), nine belong to randomized controlled trials (*n* = 596). In total the studies comprise 677 patients, 367 hereof were treated with probiotics/synbiotics. In the nine RCTs the patients in the control groups received either no treatment (*n* = 157), placebo (*n* = 80), another probiotic (Streptococcus faecalis, *n* = 34) or an antibiotic (neomycin, *n* = 22). The selected preparations are shown in Table 1. In seven studies synbiotics were given [35–37, 39–42], in two studies only probiotic strains were used [38, 43]. The duration of application varied (Table 1).

Stool examination was done by culture in five and by sequencing in five publications. The postoperative day of examination varied (first day of bowel function to the seventh or eighth postoperative day). The area of surgery was mixed: Four studies (one cohort study and three RCT) evaluated patients after colorectal surgery, one study assessed patients after esophagectomy and another one looked at patients after operation of the pancreas and bile-system. Four studies focused on liver surgery, hereof one study adressed patients undergoing living donor liver transplantation. All patients except for the patients undergoing liver transplantation were operated because of cancer.

### Applied antibiotics, bowel preparation and nutrition

All patients were treated with perioperative antibiotics. Unfortunately, only six publications reported about the kind of antibiotic treatment [38–41, 43, 44]. The antibiotics differed between the studies. One study used Ceftriaxon and Metronidazol perioperatively [38], another Cefoperazone, Sulbactam and Flomoxefuntil [40] the second postoperative day. Another study started Amoxicillin and Cefotiam perioperatively and continued the application until the fourth postoperative day [39]. Two studies applied preoperative antibiotics (Kanamycin/ Metronidazol in one and Gentamicin/Metronidazol in another one) [43, 44]. Zhang et al. continued the antibiotic application with Cefuroxime perioperatively and added another dose of Metronidazol postoperatively up to the fifth postoperative day [43]. Ohigashi et al. added a perioperative dose of Cefmetazol, which was repeated in the first 24 h after operation [44]. Seven studies performed a mechanic bowel preparation before operation [35–38, 40, 43, 44]. The preoperative nutrition is reported in six studies [35, 37, 38, 40, 42, 43]. Most of the patients had a regular nutrition preoperative [35, 37, 38, 40]. The patients in one study were set on a liquid diet two days preoperatively [43]. Postoperatively, most of the patients had a combination of enteral and parenteral feeding as a bridging until normalization of the bowel function [35, 37, 39, 40, 43]. Regular oral nutrition was initiated as soon as possible after surgery [38, 39, 44]. Only patients after esophagectomy did not start oral nutrition until the tenth postoperative day [41]. One study missed to report pre- and as well postoperative nutrition [36].

### Postoperative changing of gut microbiome

All studies reported postoperative changes of the gut flora. The amount of bacteria decreased after surgery in five studies to different degrees [40–44], but only one study found a significant reduction [41]. Three studies showed no difference between the pre- and postoperative number of bacteria [35, 37, 38] and two studies did not report the bacterial amount [36, 39].

Surgical procedures tended to result in an increase of potentially pathogenic bacteria and a decrease of Lactobacilli and Bifidobacteria [38–43]. The cohort study had a similar result as controlled studies [44]. Potentially pathogenic bacteria like Pseudomonas, Staphylococcus and Enterococci were identified as a possible source of infection in one study [44]. However, the changes of gut flora reached except of one study [41], not the level of statistical significance.

### Effect of applied synbiotics/probiotics on gut microbiome

The preoperative application of synbiotics/probiotics caused a change of gut flora before operation [35–42]. The count of Lactobacilli and Bifidobacteria were also higher on the seventh postoperative day in patients, who were treated with synbiotics [35, 37, 38, 41, 42]. The count of Bifido-bacteria was significantly higher in five studies, in which Bifidobacteria were administered [35, 38, 40–42]. Two of the five studies also reported a significantly higher number of Lactobacilli [35, 41]. One study showed a reduced number of Bifidobacterium longum in both groups in the first spontaneous fecal sample [43]. The level of reduction of Bifidobacterium longum was significantly smaller in patients treated with probiotics. Also the increase of *Escherichia coli* was significantly lower in treated group compared to control group [43]. Likewise the postoperative increase of potentially pathogenic bacteria after treatment with synbiotics was less pronounced in the others publications [35, 38, 39, 41, 42]. In contrast one study showed an opposite effect [40]: Patients after hepatic surgery had in probiotic group clearly a lower count of Lactobacilli and Bifidobacteria on the seventh postoperative day. Potentially pathogenic bacteria were similar between the groups [40]. Nevertheless, the rate of infectious complications was lower in treated group than in control group. This tendency could be shown in all studies except of one [36]. This study, however, had an alternative concept: Patients were assigned to mechanical bowel preparation (group 1–3) without additional treatment (group 1), additional treatment with neomycin (group 2), additional treatment with neomycin and synbiotics (group 3) or treatment with neomycin and synbiotics without mechanical bowel preparation (group 4). They measured just a selection of potentially pathogenic bacteria and not the whole spectrum of the gut. Only the third group had a significant decrease of Enterobacteriacea [36]. There were no differences between the groups with regard to infectious complications.

Five studies reported about systemic inflammatory response syndrome (SIRS) [36, 38, 40–42]. The inflammatory answer was little lower in synbiotic-treated groups (shorter duration of SIRS and faster recovery) in three studies (two trials with significant differences, one tendency) [40–42]. However, the other two studies showed no difference [36, 38]. The laboratory tests for inflammation failed to show a clear relation to SIRS. Four publications reported a non-significant lower count of white blood cells and C-reactive protein (CRP) or a faster recovery [35, 37, 41, 43], three reported no difference [36, 40, 42].

### Concentration of organic acids in stool and stool pH

Another parameter for controlling the effect of synbiotics/probiotics is the concentration of organic acids in stool. It was measured in six studies [35, 37, 40–42, 44]. One cohort study showed a significant postoperative decrease of organic acids after colorectal surgery [44]. Most of the patients in controlled studies had a higher concentration of organic acids in stool after treatment with synbiotics. The effect reached statistical significance in four studies [35, 37, 41, 42]. One study compared preoperative with postoperative application of synbiotics. They found a significant increase of organic acids in control group on the 21st day after operation compared to preoperative measuring [37].

pH of stool was measured in six studies [35, 37, 40–42, 44]. There were no differences of pH pre- and postoperatively in cohort study [44]. Three studies reported a lower pH for patients, who were treated with probiotics/synbiotics compared to control groups [40–42]. This difference was significant on the seventh postoperative day in two trials [41, 42]. Two RCT found no difference [35, 37].

One study also correlated amount of organic acids with number of bacteria in the gut [41]. If there were more potentially pathogenic bacteria like Enterobacter species and Pseudomonas, amount of organic acids was significantly decreased. On the other hand there was an increase of organic acids, if the number of Bifidobacteria was higher.

### Physical comfort and bowel function

Three publications reported physical comfort and bowel function of patients [38, 41, 43]. In two of three studies treatment with synbiotics/probiotics caused a significantly faster recovery of bowel movement. One study reported that first day of flatus was significantly earlier in patients with treatment [41]. Another one found a significant earlier defecation [38]. Both studies found a lower rate of postoperative diarrhea in probiotic/synbiotic-treated patients, which was significant in one study [38]. The rate of typical postoperative abdominal symptoms like cramping and distension was lower in probiotic/synbiotic-treated group [38, 41, 43], result was significant in one study [38].

### Risk of bias within the studies

The reviewed studies showed uncontrolled bias/confounding factors. Infectious complications were not clearly defined in all studies. Operations differed (four studies dealing with colorectal surgery [36, 38, 41, 43], four dealing with liver surgery [35, 37, 39, 40] and one with the pancreas/bile-system [42] and the esophagus [41], respectively). Relevant parameters were not reported or differed, if reported (e.g. blood loss, duration of operation, degree of resection, peri- and postoperative nutrition and application of antibiotics). Also applied probiotics/synbiotics differed (shown in Table 1).

Just two of the RCTs were double-blinded and placebo-controlled [38, 43]. In one study the baseline characteristics of control and intervention group differed significantly [40]. Just two studies defined a primary endpoint and calculated the sample size [36, 39]. All of the other included studies did not define primary endpoints and did multiple testing without adjustment.

## Discussion

Whether or not the gut microbiota are related to anastomotic leakage or wound infection after gastrointestinal surgery remains a highly relevant question with regard to the management of patients undergoing abdominal surgery. The results of the ten included studies suggest that there might be a relationship between the gut flora and the development of postoperative complications. The quality of the publications, however, is too low to draw firm conclusions. Every surgical procedure is a challenging situation for a human being and is associated with an inflammatory response, which depends on the surgical technique [45, 46]. It is therefore problematic to compare the effects of different operation techniques. In addition, the different operation techniques may differently influence the gut microbiome, e.g. colorectal surgery might have a higher influence on the gut microbiome than operations, which do not directly involve the gut. Although the influence of operation related parameters on the postoperative course is known, most of the reviewed studies gave only little information on operation time and exact technique [46].

The balance of the microbiome is susceptible for external factors like nutrition [2]. A well-defined pre- and postoperative nutrition is, therefore, important for studies investigating the impact of surgery on the gut flora. Most of the studies classified the preoperative and postoperative nutrition as regular. None of the studies, however, defined what this meant exactly. In addition, most of the studies failed to give detailed information about an additional postoperative antibiotic treatment.

Another problematic aspect is the constitution of the gut flora. The importance of a healthy gut microbiome became popular in the last years, but it is not clarified what kind of microbiological spectrum is healthy and what is not. Stool samples of putatively healthy subjects showed a broad spectrum of gut microbiota [3]. In the included studies the terms “harmful” and “beneficial” appeared to describe gut bacteria. For example Enterococci were often regarded as harmful, although it is known that Enterococci also have beneficial functions [47]. Because the role of different bacteria in the gastrointestinal tract is widely unknown it is also unclear, which kind of bacteria is optimal for treatment. Most of the reviewed studies applied a combination of probiotic and synbiotic preparations (Lactobacillus casei, Bifidobacterium breve and Galactooligosaccharides) [35, 37, 39–42]. The selection criteria for each combination are not reported.

Another unclear point is, whether changes of the microbiome are just an indicator for infectious complications or play a causal role. The reviewed studies used the term “infectious complication” not homogeneously and without a clear definition. Although e. g. a postoperative pneumonia could be rated as an infectious complication it is obviously not the same as a urinary tract infection or an anastomotic leakage. A few of the reviewed RCT summarized all of them and compared the results between control and treatment group. Some also added signs of systemic infection like SIRS or septicemia to the list of infectious complications. Such a method of summarizing is debatable and might blur the results.

Finally, this discussion turns to the examination of stool, which is a crucial point. The examination of stool by culture is not that meaningful, it is just a selection of the gut flora, not an evaluation of the whole bacterial diversity. In the last years PCR has been established to investigate the microflora. With this technique it has been shown, that the diversity of the flora is a deciding parameter [1, 8]. However, for the analysis of sequencing results experience is needed. The method is sensitive and the samples could easily be contaminated [48]. As a consequence of this only few laboratories have the ability for an advanced microbiome analysis. The analysis of the microbiome of the gut is still not a routine examination. This might be one more reason why the clinical evidence for a relation between gut flora and surgical complications is still not proven.

Despite a critical view on the reviewed studies is necessary and justified, the lower infection rate in patients, which were treated with probiotics or synbiotics, is promising. Eight of nine RCT showed a benefit with regards to infectious complications in patients, who were treated with synbiotics or probiotics. Only one of nine reviewed RCTs did not show the same effect on the gut flora and reported even a contrary effect [40]. This study, however, was flawed by randomization bias. The patients in the intervention group had significantly longer operation time, significantly larger blood loss during operation and larger dimension of resection. All of these factors might influence the gut microbiome and might cause contrary results. Although patients treated with synbiotics had worse operation related conditions they still had an apparently better postoperative healing than patients of the control group. Therefore this study does not contradict the assumption of beneficial effects of synbiotics.

Moreover, the results of three double-blinded RCTs indicated that the application of synbiotics or probiotics improved the postoperative bowel function. Postoperative disturbances of the bowel function are a relevant problem for patients. They can cause abdominal distension, causing nausea, vomiting and abdominal pain, which may result in a delayed oral nutrition and mobilization [49]. If modulation of the microflora could prevent these complications, it would be an improvement.

The probability of preventing postoperative complications like wound infections by modification of the gut flora, furthermore, is supported by an amount of clinical trials, which investigated the benefit of perioperative application of antibiotics. In colorectal surgery the application of antibiotics is thought to reduce the rate of postoperative wound infection by as much as 75% and is therefore a standard in colorectal surgery [23]. To overcome the gap of knowledge we suggest a prospective cohort study without probiotics/synbiotics intervention but with strict control of possible bias/confounding factors comparing patients with and without postoperative complications in regards to the microbiome diversity measured with next generation sequencing.

## Conclusion

As a result from nine RCTs and one cohort study there might be a relationship between the gut flora and the development of postoperative complications. Due to methodological shortcomings of the included studies and uncontrolled bias/confounding factors, however, there remains a high level of uncertainty. Future studies should define a primary endpoint, perform a sample size calculation and control for bias/confounding factors like treatment with antibiotics and postoperative nutrition to clarify the important issue of a correlation between gut microflora and postoperative complications.

## Additional file

Additional file 1: Whole search strategy of systematic review. Contains flowcharts, showing the detailed search strategy with search terms, includes web addresses of reference databases. (PDF 277 kb)

## Abbreviations

CRP: C-reactive protein
n. s.: not specified
RCT: Randomized controlled trial
SIRS: Systemic inflammatory response syndrome
