# Supplementary figures and images for: Postoperative changes of the microbiome: are surgical complications related to the gut flora? A systematic review
Source: BMC Surg. 2017 Dec 4;17:125. doi: 10.1186/s12893-017-0325-8 (PMC5715992; doi:10.1186/s12893-017-0325-8)

## Appendix

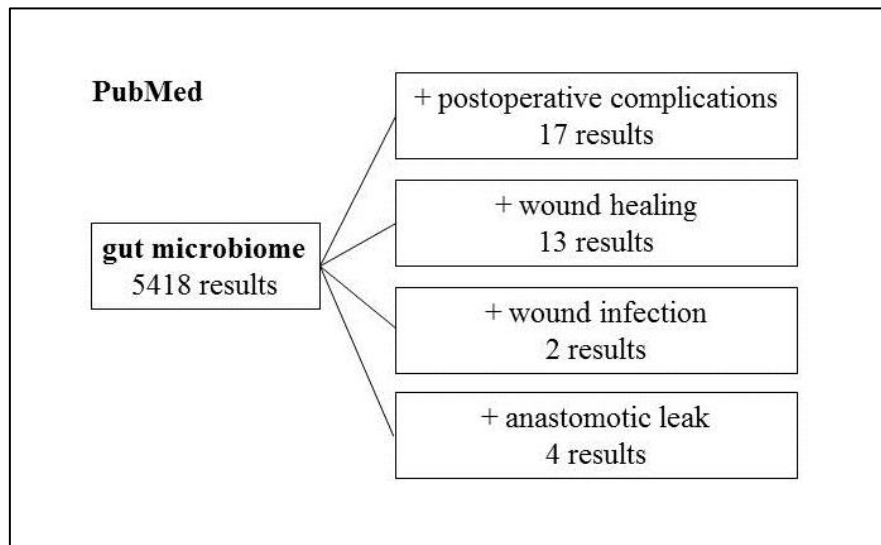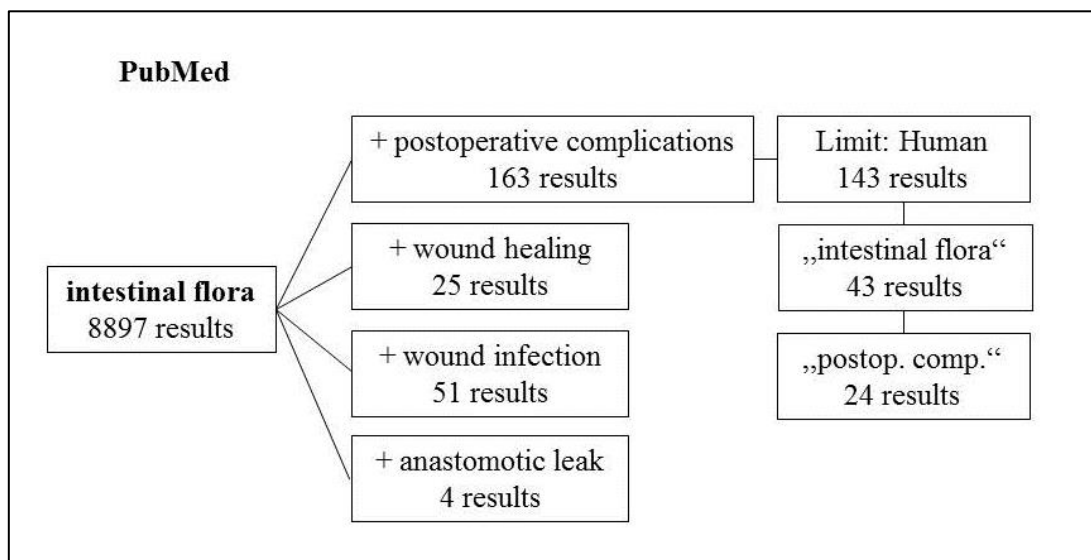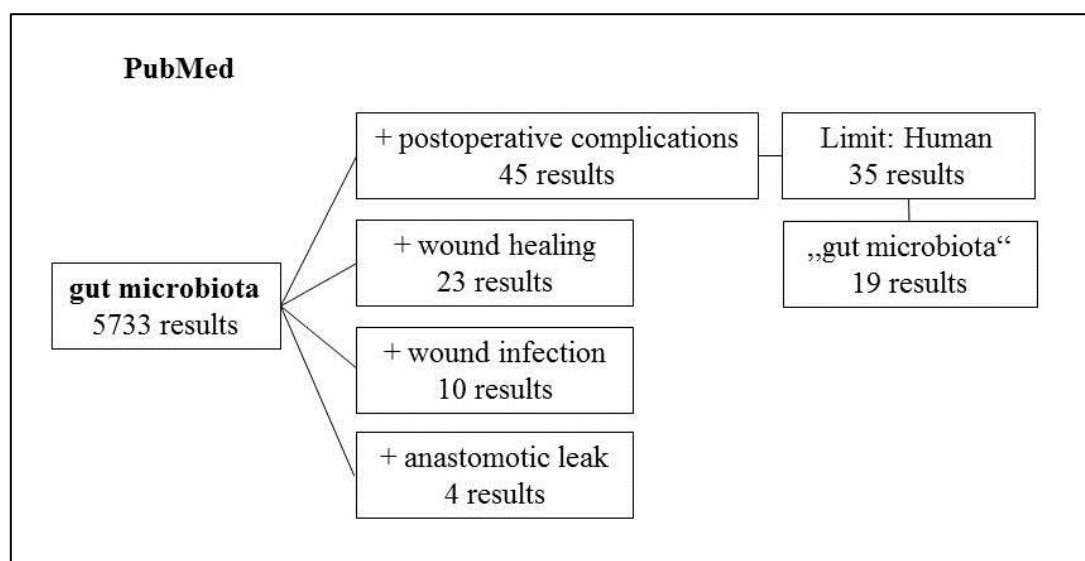

<https://www.ncbi.nlm.nih.gov/pubmed>

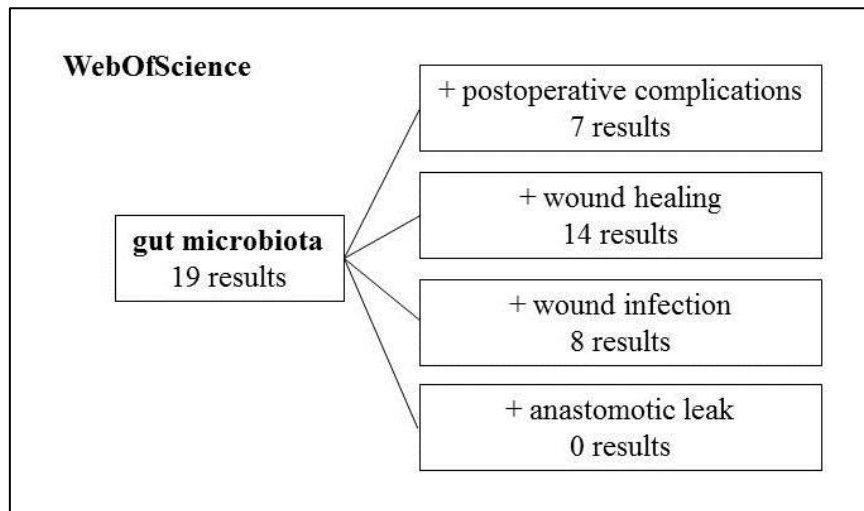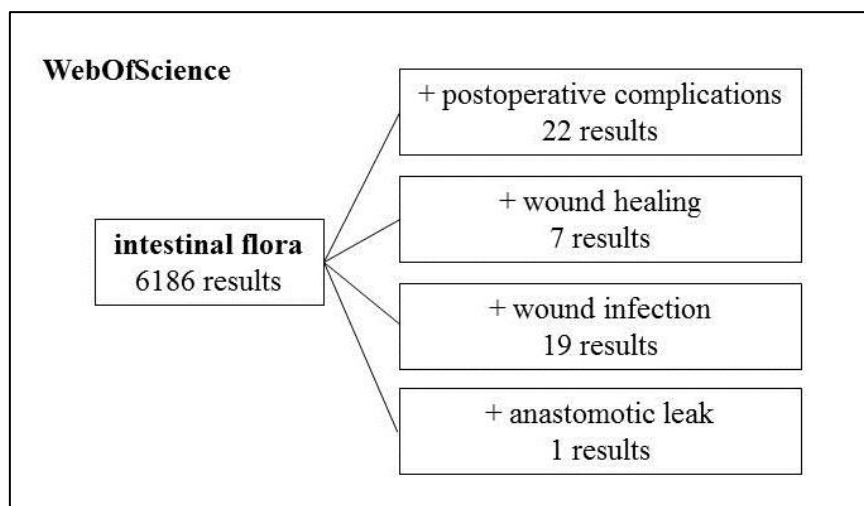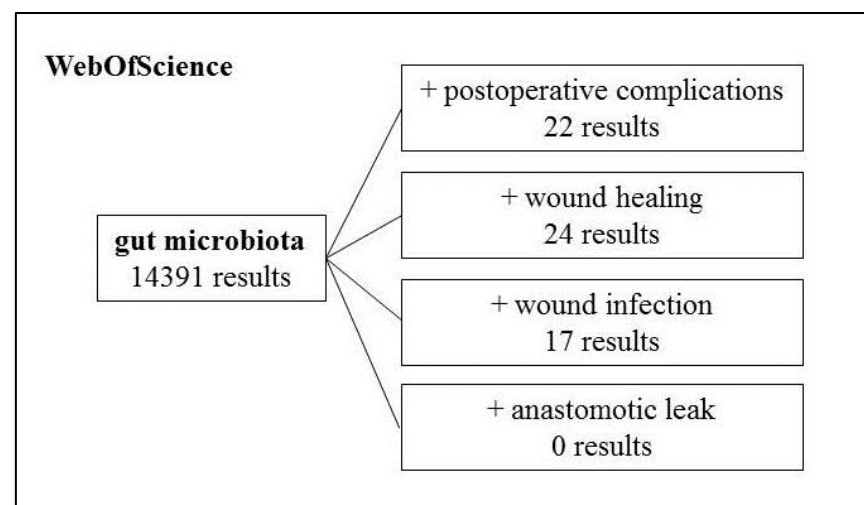

Supplement: Additional file 1: — Whole search strategy of systematic review. Contains flowcharts, showing the detailed search strategy with search terms, includes web addresses of reference databases. (PDF 277 kb) [file 12893_2017_325_MOESM1_ESM.pdf]
